# Dementia, Work and Employability: Using the Capability Approach to Understand the Employability Potential for People Living with Dementia

**DOI:** 10.1177/0950017020961929

**Published:** 2020-12-02

**Authors:** Louise Ritchie, Valerie Egdell, Michael Danson, Mandy Cook, Jill Stavert, Debbie Tolson

**Affiliations:** University of the West of Scotland, UK; Northumbria University, UK; Heriot-Watt University, UK; Forest Research, UK; Edinburgh Napier University, UK; University of the West of Scotland, UK

**Keywords:** Capability Approach, dementia, disability, employability, sustainable employability

## Abstract

The importance of remaining in, or re-entering, the labour market is emphasised by governments internationally. While this may bring benefits, progressive disabilities such as dementia affect an individual’s employability. Although employers have legal obligations to support employees with disabilities, research suggests that employers are not providing this support to employees living with dementia and are undermining their capabilities. Drawing on interview data from 38 key informants collected over two studies, we explore the potential for supporting and promoting the employability of people living with dementia. A model of sustainable employability based on the Capability Approach is used as a lens to explore this issue. The findings demonstrate the implications of progressive disabilities for employability when the worker and their family are faced with dealing with a disability in a period of uncertainty with a lack of public and workplace understanding.

## Introduction

Dementia is an umbrella term for illness caused by diseases such as Alzheimer’s disease, vascular dementia and Lewy body dementia, which are neuroprogressive and terminal. Symptoms include disturbance of functions such as memory, orientation, decision making, learning capacity and communication (World Health Organization, 2012). While the prevalence of dementia increases with age, in the UK an estimated 5% of people living with dementia are under the age of 65, out of an adult population living with dementia of 850,000 (Alzheimer’s Society, 2020). Furthermore, with the focus on extending working lives (Danson, 2007) and improved diagnosis and recognition of early-onset dementias (Robertson et al., 2015), it is likely that the proportion of employees living with dementia will increase. However, there is a paucity of research on the employability of those living with dementia.

The UK government emphasises the importance of remaining in, or re-entering, the labour market (Baumberg, 2014). Legislation and human rights standards require that disabled people are supported in the workplace through reasonable adjustments/accommodations (Egdell et al., 2018). While there are strong imperatives for individuals to remain in work (Greenwood and Smith, 2016; Öhman et al., 2001; Ritchie et al., 2018), evidence suggests that people living with dementia are not supported to do so, with negative effects for their financial, social and psychological well-being (Chaplin and Davidson, 2016; Evans, 2019; Ritchie et al., 2018; Williams et al., 2018). Evidence suggests that there are employment inequalities for people living with dementia. As a result, there is an urgent need to develop evidence-informed guidance and practical employability supports (Thomson et al., 2019) to promote understanding of the workplace issues facing employees living with dementia for employers, families, health and social care professionals and support service providers. A theoretically informed understanding of the factors which influence the employability of persons living with dementia is needed. In this article, we develop theoretical insights in tandem with an applied understanding of dementia in relation to employability. Recognising dementia as a disability (Gove et al., 2017), and using the sustainable employability model (Van der Kink et al., 2016), we re-examine interview data from two recent UK studies. This rigorous approach deepens understanding of the factors shaping the employability of people living with dementia, which is a prerequisite to the development of evidence-informed employment support.

## The employment experiences of persons living with dementia

Although research on dementia and employment is sparse, the small body of evidence suggests that people living with dementia can and do continue working post-diagnosis. However, support can be complex to manage and may require multi-agency input (Ritchie et al., 2018), and employment type, organisational size and ethos influence opportunities to continue employment **(**Egdell et al., 2019 **)**. Several studies report that people do not continue working after diagnosis, despite individuals feeling that they retain skills and experience to contribute to the workplace (Chaplin and Davidson, 2016; Evans, 2019; Ritchie et al., 2018; Williams et al., 2018). This is at odds with efforts by the dementia-friendly movement to create supportive and inclusive environments to promote dignity, empowerment and autonomy (Hebert and Scales, 2019).

Labelling of people living with dementia as ‘poor workers’ is a common theme across the literature (Evans, 2019). Williams et al. (2018) highlight the stigma and discrimination faced in becoming ‘a person living with dementia’ in the workplace, as an individual goes from being a competent, well-respected colleague to being viewed as incompetent and lacking autonomy. It is not unusual for people with early symptoms to be made redundant or dismissed for incompetence (Bentham and La Fontaine, 2007). ‘Social invisibility’ in the workplace, excluding employees from meetings and covert observation of performance perpetuate the ‘poor worker’ label, which undermines employees’ well-being and confidence and leads them to question their worth and future employment (Chaplin and Davidson, 2016; Williams et al., 2018).

Framing dementia as a disability results in a focus on supporting people living with dementia to be effective citizens (Bartlett, 2014). In an employment context, linking dementia and disability due to the substantial and long-term adverse effects on the individual’s ability to carry out normal day-to-day activities enacts the UK’s equality legislation (HM Government, 2010). However, research shows that employers may not recognise this (Egdell et al., 2019). Additionally, in the UK there is no legal case history to demonstrate the application of these laws to protect employees living with dementia (Egdell et al., 2018). Employers often initiate decisions to leave work; failing to make and/or consider reasonable adjustments (Chaplin and Davidson, 2016; Evans, 2019; Thomson et al., 2019; Williams et al., 2018).

## Sustainable employability from a Capability Approach

Employability is a multi-dimensional and complex concept related to the ability of employed and unemployed individuals to move into or within employment (McQuaid and Lindsay, 2005). In this article, we apply Van der Klink et al.’s (2016) model of sustainable employability, based on Sen’s Capability Approach (CA), to understand the employability of people living with dementia. While the sustainable employability model is applied to understand the experiences of disabled workers (e.g. those with multiple sclerosis (Van Gorp et al., 2018)), we are unaware of any application to understand the employability of people living with dementia to date.

While the CA originates in welfare economics, it is increasingly applied to complex and insecure labour market transitions in sociological research (Beck, 2018; Egdell and Beck, 2020). The CA focuses ‘directly on freedom as such rather than on the means to achieve freedom, and it identifies the real alternatives we have’ (Sen, 2003: 49). While ‘achievement is concerned with what we manage to accomplish’, freedom is concerned ‘with the real opportunity that we have to accomplish what we value’ (Sen, 2003: 31). Thus, the CA moves beyond resourcist approaches that prioritise ‘means’ of freedom (i.e. Rawls’ (1999) principle of justice) to pay attention to ‘extents’ of freedom (Sen, 2003, 2009). Interpersonal variations in the transformation of primary goods into capabilities are acknowledged. It considers the means available to individuals, as well as what individuals do and are (their functionings), and all that an individual can do or be (the individual’s capability-set), accounting for wider structural characteristics (‘conversion factors’) that may affect the transformation of resources into capabilities. As such, while individuals may have the same primary goods, they may have different capability-sets (Sen, 2003, 2009).

The CA is critiqued for not addressing injustice inherent in capitalism (Dean, 2009) – links can be made here to arguments that the exclusion of disabled people is rooted in capitalism (Oliver and Barnes, 2012). Nevertheless, the CA offers a distinctive approach, with the focus on capability-sets setting it apart in framings of welfare and well-being (Egdell and Beck, 2020) and some (e.g. Carpenter, 2009) argue that a radical CA could be developed through connections with fuller political economic/social analysis. Framing employability from a CA draws attention to employment possibilities, social environments, employers and individuals (Leßmann and Bonvin, 2011), allowing us to develop a contextualised understanding of the labour market experiences of people living with dementia. It also draws attention to what is involved in securing rights (Nussbaum, 1997). It recognises that, while legislative standards in principle require that people living with dementia are afforded employment rights, it does not remove the possibility that their employability is constrained by a range of complex and intersecting factors (Ritchie et al., 2018). While some argue that the CA overemphasises rational cognitive action and does not substantively discuss processes through which decisions are made (Dean, 2009; Gasper, 1997; Zimmerman, 2006), Sen (2009) does suggest public deliberation in the development of capabilities ‘lists’. Equally, while the impact of deprivation on choice reasoning processes is not well developed in the CA, it does highlight that individuals who are persistent victims of discrimination may be constrained in their choices because of internalised conceptions of their own unequal self-worth (Egdell and Beck, 2020; Nussbaum, 1997). Thus, it provides a lens to understand the complex relationship between economic activity, employability and disability at the individual and labour market level (Anyadike-Danes, 2010; McQuaid and Lindsay, 2005).

Van der Klink et al.’s (2016) model of sustainable employability based on the CA acknowledges the complexity of what constitutes functioning in work (Fleuren et al., 2016). It requires that:

Throughout their working lives, workers can achieve tangible opportunities in the form of a set of capabilities. They also enjoy the necessary conditions that allow them to make a valuable contribution through their work, now and in the future, while safeguarding their health and welfare. This requires, on the one hand, a work context that facilitates this for them and on the other, the attitude and motivation to exploit these opportunities. (Van der Klink et al., 2016: 74)

The model posits that work should be a valuable part of the life-course (Abma et al., 2016). Employability should be adaptable and intrinsically linked to the individual’s capabilities and the work environment (i.e. they are able and enabled). It reflects the process where workers convert resources into opportunities to achieve goals that they value (e.g. the opportunity to develop knowledge and skills); as well as the personal inputs (i.e. personal capacity) and work inputs (i.e. work characteristics). The role of personal and contextual conversion factors (e.g. individual motivation and organisational policy) which enable or constrain potential opportunities are accounted for (Van der Klink et al., 2016: 74–75). Van der Klink et al. (2016: 74) highlight that the ‘crux of the capability concept lies in the combination of various meanings of “can”’. This premise provides our analytical framework: (1) being able to work (i.e. personal and work resources); (2) having the opportunity to work (i.e. material resources); and (3) being facilitated and allowed to work (i.e. physical and social environment).

## Methods

In this article, we present a secondary analysis of interview data with key informants collected over two studies (Table 1). Both datasets were previously analysed as key components of the original research studies to understand the employment context of people living with dementia. For this article, secondary qualitative analysis was undertaken, bringing together the key informant datasets. Secondary analysis entails using pre-existing qualitative data to address new and/or further research questions or to apply theoretical frameworks not used in the original study (Heaton, 2008; Notz, 2005; Sherif, 2018).

**Table 1.** Overview of studies 1 and 2.

	Study 1	Study 2
Date	2013–2015	2016–2018
Funder	Alzheimer’s Society	Carnegie Trust for the Universities of Scotland
Study aim	To explore the employment-related experiences of people with dementia or mild cognitive impairment (MCI), and attitudes of employers and/or co-workers towards supporting people with dementia, in order to identify the potential for continued employment post-diagnosis.	To explore the policies, practices and attitudes of employers in Scotland with regards to dementia in the workplace in order to determine the extent to which they are meeting their legal, equality and human rights duties and set out any possible adaptions that can be made.
Research team (authors’ initials)	LR, DT, MD	JS, VE, LR, DT, MD, MC
Geographical location	UK	Scotland
Reference	Ritchie et al. (2018)	Egdell et al. (2019)

Both studies explored dementia in the workplace: Study 1 (S1) focused on the experiences of employees living with dementia; while Study 2 (S2) explored employers’ perceptions. In the initial phase of both studies, key informants believed to have some experience or relevant expertise (Marshall, 1996) were interviewed to understand the employment context for people living with dementia and the support available to them and their employers. These interviews were not intended to offer the voice, views and opinions of people living with dementia, but drew on the key informants’ work-related experiences of working with people living with dementia. Therefore, in this article, we do not focus on direct employment experiences. Rather, the opinions, knowledge and understanding of key informants who support the employability of those living with dementia are focused upon, providing tentative insights into the issues facing people living with dementia in the workplace.

### Study 1

In S1, semi-structured interviews were conducted with 19 key informants working in different parts of the UK (Table 2). Purposive sampling was used, with potential participants identified by the research team and the project advisory group based on previous contacts and organisations with relevant expertise. The interviews explored the current environment for those living with dementia in terms of employment policy and practice; and the role of voluntary organisations, statutory agencies, employers’ organisations, and trade unions. Interviews were conducted face-to-face and audio-recorded and transcribed with the participants’ permission. Ethical approval was granted by West of Scotland Research Ethics Service (WoSREC) (approval number 13/WS/0145).

**Table 2.** Study 1 sample characteristics.

	Role	Expertise
1	Policy-maker	Government policy relating to dementia
2	Healthcare professional	Dementia, psychiatry, diagnosis
3	HR professional	Employment policy and practice
4	Healthcare professional	Early-onset dementia, nursing, supporting people to continue working
5	Healthcare professional	Dementia, neuropsychology
6	Healthcare professional	Mental health vocational rehabilitation, occupational therapy
7	Healthcare professional	Dementia, patients who have been/are in employment, psychiatry
8	Healthcare professional	Early-onset dementia, patients who have been/are in employment, psychiatry
9	Support organisation representative	Disability, employment, government policy and benefits
10	Support organisation representative	Employer support, occupational health
11	Healthcare professional	General practice, diagnosis
12	Support organisation representative	Dementia, supporting people to continue working
13	Person with dementia	Experience of being in employment when diagnosed with dementia
14	Trade union representative	Employee support and disability specialist
15	HR professional	Disability and employment
16	HR professional	Employment policy
17	Policy-maker	Government policy relating to dementia
18	Trade union representative	Employee support, employment policy and practice
19	Healthcare professional	Psychology, dementia, diagnosis

### Study 2

In S2, semi-structured interviews were conducted with 20 Scotland-based key informants (Table 3). Purposive sampling was used, with the research team drawing on existing links and those of the study’s advisory group. Snowballing techniques were also used. The purpose of the interviews was to gauge expert views on the experiences of people living with dementia in the workplace, the difficulties they might encounter and employer policy and practice. Interviews were conducted face-to-face and were audio-recorded and transcribed with the participants’ permission. Ethical approval was granted by Edinburgh Napier Business School Research Integrity Committee (ref. ENBS/2016-17/007).

**Table 3.** Study 2 sample characteristics.

	Role	Expertise
1	Healthcare professional	Supporting people living with dementia to continue in employment
2	Healthcare professional	Early-onset dementia
3	Healthcare professional	Health and well-being in the workplace
4	Healthcare professional	Supporting people living with dementia to continue in employment
5	Healthcare professional	Supporting people living with dementia to continue working / reasonable adjustments
6	Healthcare professional	Dementia / supporting people living with dementia to continue working
7	HR professional	Employment policy
8	HR professional	Employment policy
9	Lawyer	Employment / mental health / incapacity law
10	Lawyer	Employment / mental health / incapacity law
11	Person living with dementia	Experience of being in employment when diagnosed with dementia
12	Policy-maker	Government policy relating to dementia
13	Support organisation representative	Supporting volunteers living with dementia
14	Support organisation representative	Employer dementia awareness
15	Support organisation representative	Employer dementia awareness
16	Support organisation representative	Supporting people with mental health conditions
17	Support organisation representative	Dementia / supporting people to continue working
18	Support organisation representative	Disability rights and support in the workplace
19	Support organisation representative	Employer awareness of the needs of older workers
20	Support organisation representative	Employment / health / communities in later life

### Secondary data analysis

To comply with ethical requirements, transcripts were analysed within the original research teams. In the re-analysis, both datasets were coded using Framework Analysis (Ritchie and Spencer, 2002) using the framework of Van der Klink et al.’s (2016) sustainable employability model: (1) being able to work; (2) having the opportunity to work; and (3) being facilitated and allowed to work. Pertinent data extracts were identified. Once indexed, data segments were extracted and charted in a matrix. The final stage of mapping and interpretation of the data involved pooling the two matrices. Similarities and differences in the themes across both datasets were explored. An iterative process of checking, refining and defining themes until a clear and coherent representation cutting across both datasets was undertaken.

## Findings

All the respondents agreed that a diagnosis of dementia would affect an individual’s employability; however, the extent of this varied. As described above, the findings are presented in line with Van der Klink et al.’s (2016) sustainable employability model which encapsulates the interrelated building blocks of an individual’s capability-set (Sen, 2003).

### Personal and work resources – ‘being able to’

Personal resources refer to personal capacity, knowledge and ability, while work resources encompass job requirements and demands (Van der Klink et al., 2016). Thus, ‘being able to’ refers to the work that a person living with dementia has the capacity, knowledge and ability to do in the context of the job’s requirements.

While dementia is commonly perceived as a cognitive deficit (Hamilton-West et al., 2010), participants, particularly from health backgrounds, highlighted the range and variation of symptoms experienced:

It depends on what it is, what is the type of dementia, and how far they are in terms of their diagnosis . . . what the symptoms are and how it impairs a person, what are the things that they can still do, what are the things that they are good at. (Healthcare professional, S1)

Participants detailed that the different symptoms presented challenges for supporting employability, with interaction of symptoms and a person’s job (i.e. work resources) influencing ability to continue working in that environment. The participants provided examples of problems that people living with dementia had in employment because of their symptoms:

We’ve got a chap at the moment . . . the work he says is incredibly repetitive, I mean it’s the same thing day after day, week after week, month after month, he’s doing the same types of thing, in the same order . . . he says now he finds himself, he’s going up to his [boss] and saying, ‘What is it I’ve to do here?’. (Healthcare professional, S1)

In this situation, participants detailed that simple adjustments (e.g. providing standard operating procedures with step-by-step guidance for tasks) could not redress these problems. Participants felt that for employers to consider adjustments, they needed to understand the personal resources of the individual employee living with dementia: ‘using people’s assets within the workplace, and not focusing on the stuff they can’t do, because there’s stuff that everybody can’t do’ (Healthcare professional, S2). However, participants argued that employers did not perceive employees living with dementia as skilled and experienced, and they did not meet legislative obligations to make adjustments. Rather, employees living with dementia were framed as a ‘risk’, further affecting perceptions regarding ability:

Someone in a [lab] where there are actual hazards to health and safety . . . obviously, we would need to be looking at whether their retention of what they needed to do or know was sufficient for them to be safeguarded. (HR professional, S1)

Another personal resource identified that could affect the success of workplace adjustments or supports was people living with dementia’s insight into their abilities. This could affect their capacity to access and maintain support, as well as challenge their sense of self as a worker:

I discovered that I was forgetting things and I didn’t know I was forgetting things. And people would say, ‘Have you done so and so?’, and I would think he didn’t ask me . . . I couldn’t believe it that I’d said I’ll do this for you and then I hadn’t done it . . . I didn’t remember I’d promised to do it for them. (Person living with dementia, S1)

Previous research shows that insight into abilities and the impact of symptoms on performance may help employees to accept that to continue working they will have to make changes, but that this does not necessarily mean the end of their working lives (Evans, 2019; Ritchie et al., 2018; Williams et al., 2018):

He’s been given kind of lighter, less stressful duties, which he’s accepted and he’s happy with it. Because he’s now working full-time again. (Healthcare professional, S1)

Participants highlighted the distinction between *not being able to do a job* and *not being able to work*. An individual may be able to continue working in another or adapted role. Thus, participants demonstrated that work-specific assessments, capturing role and work demands, were required. However, while individuals may have the skills and experience to continue employment, this was not always appreciated by employers or healthcare professionals, undermining the personal resources of people living with dementia:

Her consultant had told her you cannot continue to do your job . . . [she] was very upset by that, and also questioned whether that consultant understood what her job actually entailed, and how her impairment might or might not impact on her doing that particular job. (Support organisation representative, S2)

Some participants emphasised that there should be a focus on ability, not disability. The expectation that people living with dementia could not carry on working was countered by examples where individuals had taken up voluntary, casual or seasonal work. These examples demonstrated that people living with dementia had the capacity, knowledge and ability to be employable:

A man, who at his diagnosis was told by the consultant that he should give up his job, 3 months later then became a volunteer . . . seemed strange that somebody would be asked to give up work, and then be provided with work. (Support organisation representative, S2)

Participants also noted that people living with dementia did not lose their abilities ‘overnight’, nor ‘that all the skills that that person has carried throughout the whole of their lives are diminished’ (Support organisation representative, S2). However, the importance of not setting up individuals to fail when supporting continued employment was highlighted. The challenges of taking on new roles should not be underestimated in terms of preserving dignity:

To put them into something different is almost an additional challenge, not to mention how it is for any of us when we start new jobs. It’s unfamiliar, it’s difficult, and not always that comfortable. (Healthcare professional, S2)

Participants detailed that from the perspective of an individual living with dementia, deciding to stop working required a level of insight:

I’ve had a pretty good life and I’ve always been fairly well thought of, that’s self-evident from my career . . . I don’t want to be the [person] who, you know, lost it, so, so I stopped [working]. (Person living with dementia, S1)

When exploring the personal resources of people living with dementia, the findings reflected the existing literature. People living with dementia potentially have the personal resources to continue working (Ritchie et al., 2018). However, in terms of supporting their employability, the challenge lies in recognising the personal resource, both for the individual and the organisation they work in. Support should provide a shift in focus from what they are unable to do, to what they can do. Yet, this is not always the case in practice.

### Material resources – ‘having opportunity to’

While an individual living with dementia may have the capacity, knowledge and ability essential for continued employment in a specific role, if they do not have access to, or cannot make use of, material resources, then they cannot continue employment (i.e. they do not have the opportunity to) (Van der Klink et al., 2016). This refers to the interaction with the context that enables people living with dementia to use their resources and capacities and realise opportunities for continued employment.

Participants viewed diagnosis as unlocking support – although not in all instances – as it offered insights into progression and potential adjustments. They detailed that diagnosis can take several years for those with early-onset dementia (see also Greenwood and Smith, 2016; Millenaar et al., 2016). Those who did not have the material resource of a diagnosis could be perceived as an incompetent worker and lose their job (see also Evans, 2019; Thomson et al., 2019), with no recourse once a diagnosis was received:

They have to kind of rule out lots of different things first, you know – it’s not the automatic thing you would think of for somebody, for instance, like who’s 49 – so I think for some of them it can take over a year for diagnosis, by which time, you know, things are probably quite bad at work, I think – if they’re still in work. (Support organisation representative, S1)

We do know a lot of people who have developed dementia in the workplace and because of the symptoms, lost their job, without having had a diagnosis, and then they’re out. They then get a diagnosis and they have no recourse. (Support organisation representative, S2)

Participants discussed how clinical and support services may be inaccessible to those without a diagnosis and/or those aged under 65. Persons living with dementia may have left work by the time they were referred onto support services, and therefore deprived of employability support:

All the way through the process we were waiting on the formal diagnosis, and [the person living with dementia] lost [his/her] job whilst the process was going on. We tried to look at pursuing other employment, but it was difficult because occupational health services wouldn’t have conversations around dementia, because they didn’t have the diagnosis. (Healthcare professional, S2)

In both studies, participants were asked whether further support services needed to be developed and whether the legislative framework adequately supported individuals (i.e. providing opportunities to work and be supported). The general perception was that there was no need to develop specific services and policies to support employees living with dementia. Instead, better use of existing facilities was needed to ensure multi-disciplinary support. The majority felt that employees living with dementia should be recognised as part of an employer’s general disability policy:

It doesn’t matter whether it’s multiple sclerosis, . . . or they’d become physically disabled in some way . . . Basically, the job of the line manager is to understand the impact of the colleague’s condition and then what steps can be taken to mitigate the impact . . . I would say we would do exactly the same thing for a colleague who develops dementia. (HR professional, S1)

However, it was acknowledged that there were employer policies and practices that could disadvantage employees living with dementia. For example, if an employee was framed as a ‘poor worker’ (see Evans, 2019; Thomson et al., 2019) then performance management could exit them from the workplace:

There is a conflict there with some workplace policies, such as sickness absence management, and performance improvement, where somebody who has had a diagnosis of dementia, as the condition progresses then clearly there may well be aspects of their role that they can no longer carry out . . . Quite often, particularly around performance improvement, employers don’t take these conditions into account. (Support organisation representative, S2)

Similarly, it was felt that while the current legislation ensured that employees living with dementia should be supported in the workplace, it was not put into practice:

Once implementation becomes a reality rather than a tick in the box that goes not much further . . . the solution to the issues are very often not to throw more laws at them, but it’s to get full implementation of the laws and international standards that already exist, and get a buy into them. (Lawyer, S2)

However, participants felt that differences in types of employment and the individual’s own experiences needed to be accounted for. It was recognised that employers had different resources available to them; with larger organisations more able to make adjustments, change roles and provide support for employees living with dementia:

A small organisation might struggle more, but adjusting the job might be possible, and in a large organisation I would hope it would always be possible to find a job that somebody might be able to do. (Support organisation representative, S2)

Nevertheless, it was highlighted that smaller organisations would have strengths in supporting an employee as well, due to the smaller teams and the closer working relationships: ‘smaller employers tend to have been much more supportive environments because people are known better’ (Support organisation representative, S1).

It was stressed by participants that employee support was available (e.g. from occupational health and trade unions), although this will vary between organisations. Additionally, they emphasised that employers, and particularly line managers, required support and information as well. There was recognition that they should not be expected to manage alone nor be expected to diagnose employees:

It’s not [the line managers’] job to diagnose. Their job is to identify the barriers and help remove the barriers. Obviously, there’s a duty of care as well, so encouraging the colleague to seek diagnosis could be beneficial. (HR professional, S1)

Participants felt that better use needed to be made of existing support services and/or legislative frameworks and argued for employer-specific advice. However, as emphasised by Williams et al. (2018), these services may not be dementia-informed or accessible to those living with early-onset dementia, highlighting the need for increased awareness around dementia and employment.

### Physical and social environment – ‘being facilitated to’

Personal and work resources are not mere determinants of the sustainable employability of persons living with dementia. They are factors that can lead to a set of potentials (the capability-set) to achieve valuable work functioning, provided that appropriate conversion factors are present (Van der Klink et al., 2016).

Participants identified that stigma and lack of understanding from others can prevent continued employment. They felt that automatic assumptions were made about the abilities of employees living with dementia: ‘the crux of this as well is making assumptions about what people are capable or not capable of without the knowledge of doing so’ (HR professional, S2). Participants felt that employers did not frame dementia as an issue that they needed to be aware of, thus they may not have recognised their legal obligations:

It’s almost like, for employers, ‘You what, you want us to tell you about dementia and how we’re going to accommodate it? Are you for real?’ That might not be what they say publicly, but in their heads. (Healthcare professional, S2)

Participants detailed that the stigma or assumptions about the capabilities of an employee living with dementia may undermine personal and material resources, and consequently, the employability of people living with dementia may not be realised. One reason for this is that people with a diagnosis may not disclose their diagnosis: ‘People think that . . . if they disclose that then they will be out – out of a job’ (Trade union representative, S1). Additionally, participants felt that a lack of understanding and awareness of dementia and its impact on people who are still working meant that if someone did disclose a diagnosis there may not be a clear plan of how to support them.

The line manager relationships; how well line managers know their employees; how line managers respond to and recognise the needs of employees; how line managers translate organisational policy on the ground; and how line managers understand reasonable adjustments – were all highlighted by participants as an important conversion factor (i.e. converting material resources into capabilities):

They would know when something is not right, they would know when things are changing, and they would know where performance or ability is being impaired . . . we do have a duty of care as an employer to our employees, and so I would like to think that you’re able to identify that as their line manager. (HR professional, S2)

Reasonable adjustments generally are, and they are, very subjective, and they rely on the goodwill of the managers and the colleagues around the person too. (HR professional, S1)

As identified in the quote above, participants expressed that support for a person living with dementia in employment needed to go beyond organisational policy and line management; colleagues also had a role to play. This was identified by a key informant living with dementia when talking about their voluntary role:

I do need the support in this of my team, like particularly the secretary . . . so I write my own agenda and we usually have a meeting beforehand and we go through exactly how we’re going to play the meeting . . . . (Person living with dementia, S1)

Other participants discussed situations where conflicts had arisen between employees living with dementia and colleagues. This underlined that the role of colleagues in creating a supportive social environment should not be understated:

We’ve certainly had a few people who have come to blows with colleagues . . . the colleagues maybe think they’re actually being obstructive or just stubborn, things like that, when in actual fact they’ve developed dementia but nobody knows. (Healthcare professional, S1)

The importance of an open and supportive workplace to the employability of persons living with dementia was highlighted, and the need to develop a culture in the spirit of the legislation:

It comes down to – do they go to the HR person and say, ‘please help me’, or does a colleague say to HR, ‘this is happening, I want you to know about it’. Or does nobody say anything to anyone . . . that’s where I think this culture thing comes in. If there is a culture of communication, people about themselves and people about others, which is clearly seen as a non-condemnatory process. (Lawyer, S2)

In relation to employers’ legal obligations, differences between employer and/or employment sectors were identified as what is viewed as ‘reasonable’ and could be context-dependent: ‘What’s reasonable will depend on the size and resources of the organisation, and how practical and effective the adjustments would be if costs are incurred’ (Policy-maker, S2). Thus, not all employees living with dementia would be able to harness the opportunities offered by legislation.

The participants provided some evidence of ‘adaptive preference’ formation – a term used in the CA literature to describe how individuals may adjust their expectations downwards (Nussbaum, 1997). It was detailed that people living with dementia may not believe in their abilities, viewing a diagnosis of dementia as signalling the end of their working lives; a perception mirrored by those around them. Participants expressed that people living with dementia might not frame dementia as a disability and, therefore, they may not see the relevance of the legislation and/or disability policies to support their continued employment:

I don’t mind being seen as a person who has dementia, but if you have a disability, in my mind, it’s somebody who is quite severely physically disabled . . . it’s not a term that sits well with me. I just say that I’ve got dementia and get on with it. (Person living with dementia, S2)

In exploring the physical and social resources, factors that could lead to a set of potentials for people living with dementia to achieve valuable work functionings were revealed. Employer, line manager and colleague awareness of dementia, legal obligations and ways in which employees living with dementia can be supported were highlighted as important conversion factors. At the same time, people living with dementia may not believe in their capacity and/or see the relevance of legislation to support their continued employment.

## Discussion and conclusions

In this article, we develop a theoretically informed understanding of employability for people living with dementia using the lens of Van der Klink et al.’s (2016) model of sustainable employability based on the CA. We show that while people living with dementia have the personal resources to work (the ability to), employment policy/practice and access to support services may undermine their abilities and compromise their employability. That is, access to material resources to support employability, and the physical and social environment (conversion factors) in which people living with dementia operate in, can impinge the individual’s ability to continue working or to seek alternative employment. These findings contribute to the small, but developing, literature-base, providing insight into how and why people with dementia are not afforded employment opportunities post-diagnosis. They also develop the sustainable employability literature in applying it to understand the employability of people living with dementia.

We demonstrate the value of a holistic framing of employability through the application of the sustainable employability model based on the CA (Van der Klink et al., 2016). This model encapsulates interrelated building blocks of an individual’s capability-set: the means to achieve (personal and work inputs); personal conversion factors (skills, knowledge and motivations that affect an individual’s capacity to transform inputs into capabilities); and external conversion factors (social and structural factors that may affect the conversion of resources into capabilities) (Van der Klink et al., 2016). While the conceptual and theoretical limitations cannot be ignored (Dean, 2009; Gasper, 1997; Zimmerman, 2006), in applying this model, attention is drawn to how the employability of people living with dementia is constrained by a range of factors, and the intersections between employability and disability (Anyadike-Danes, 2010; McQuaid and Lindsay, 2005; Van der Klink et al., 2016).

All participants agreed that a diagnosis of dementia would affect an individual’s employment; however, the extent of this varied. The capacity, knowledge, insights and ability of the individual was stressed – although this needed to be set in the context of their job requirements and demands, as well as the type of dementia and the characteristics of the organisation they work for. The importance of an open and supportive workplace was another conversion factor cited as key in facilitating continued employment, as well as relationships with, and attitudes of, line managers and colleagues. The findings illuminate how stigma and lack of understanding of dementia can impact on employability at the societal, organisational and individual level. Employers may not fully consider adjustments to support the continued employment; healthcare professionals may advise individuals to leave work; and individuals themselves may not realise their abilities because of ‘adaptive formation’ responses (Nussbaum, 1997). This questions the extent to which dementia is accepted as a disability in an employability context.

Our findings question the extent to which dementia is understood as a disability in a way that the individual is framed as ‘able to’ make a meaningful contribution through continued employment and access the supports to facilitate this. Age at diagnosis is an important factor in this context. A diagnosis when of ‘working age’ is unexpected, compromises the individual’s sense of self and well-being, and has implications for service access. This reflects previous research findings (Evans, 2019; Greenwood and Smith, 2016; Millenaar et al., 2016; Ritchie et al., 2018; Williams et al., 2018). Our findings illuminate and begin to explain barriers to employability. Williams et al. (2018) describe the experience of fighting to be recognised as a person with ability within the workplace following a diagnosis of dementia and challenging the perceptions of others. This reflects many of the key informant views that we present. They highlighted that underlying attitudes and responses to employees living with dementia (many of which could be perceived as oppressive or discriminatory) lay in a lack of education, awareness and access to supports which could enable employees living with dementia and their employer to promote employability.

There are limitations to acknowledge. Firstly, we draw on secondary data analysis. The way in which qualitative research is tailored can make it difficult to repurpose data, creating issues with ‘data fit’ in subsequent analyses (Heaton, 2008; Sherif, 2018). However, there is also an argument for not squandering rich data (Notz, 2005; Sherif, 2018). In this case, there was a clear avenue for fruitful supplementary analysis. Secondly, while we focus on the UK context, the arguments are internationally relevant. Finally, the experiences and perceptions of people living with dementia are core to understanding their employability. Although the direct experiences of two persons living with dementia are included in this article, more representative research is needed. The focus here has been understanding the context of employment. In this article, the voice of health professionals, HR professionals, lawyers, policy-makers and support organisations is stronger. However, Ritchie et al. (2018) show the importance of multi-agency support for employees living with dementia, and as such this article contributes to understanding support requirements.

It is clear from these findings that people living with dementia can continue employment; however, these capabilities are not fully explored in the current employability context. There is a need to develop and enhance existing multi-disciplinary supports to ensure that people living with dementia can recognise their own personal resources, access the material resources they need to continue employment, and ensure the physical and social environments in which they do this enable these resources to be converted into positive outcomes for employability.
